# (*E*)-*N*′-(5-Chloro-2-hydroxy­benzyl­idene)-4-(8-quinol­yloxy)butanohydrazide monohydrate

**DOI:** 10.1107/S1600536809023733

**Published:** 2009-06-27

**Authors:** Jian Zhang, Guo-Lun XiaHou, Sheng-Sen Zhang, Jing Zeng

**Affiliations:** a College of Pharmacy, Gannan Medical University, Ganzhou, Jiangxi 341000, People’s Republic of China

## Abstract

The crystal of the title Schiff base compound, C_20_H_18_ClN_3_O_3_·H_2_O, was twinned by a twofold rotation about (100). The asymmetric unit contains two crystallographically independent mol­ecules with similar conformations, and two water mol­ecules. The C=N—N angles of 115.7 (6) and 116.2 (6)° are significantly smaller than the ideal value of 120° expected for *sp* ^2^-hybridized N atoms and the dihedral angles between the benzene ring and quinoline ring system in the two mol­ecules are 52.5 (7) and 53.9 (7)°. The mol­ecules aggregate *via* C—Cl⋯π and π–π inter­actions [centroid–centroid distances = 3.696 (5)–3.892 (5) Å] and weak C—H⋯O inter­actions as parallel sheets, which are further linked by water mol­ecules through N—H⋯O and O—H⋯O hydrogen bonds into a supra­molecular two-dimensional network.

## Related literature

For background to the rational construction of new matallosupramolecular architectures, see: Muraoka *et al.* (1998 ▶); Cai *et al.* (2003 ▶); Pallavicini *et al.* (2007 ▶). For the use of 8-hydroxy­quinoline and its derivatives as ligands in this area, see: Chen *et al.* (2005 ▶); Park *et al.* (2006 ▶); Karmakar *et al.* (2007 ▶). For related structures, see: Xu *et al.* (2002 ▶); Zhang *et al.* (2005 ▶); Wen *et al.* (2005 ▶); Wei *et al.* (2004 ▶); Zheng, Li *et al.* (2008 ▶); Zheng, Wu, Lu *et al.*, (2006 ▶); Zheng (2006 ▶); Zheng, Qiu *et al.* (2006 ▶); Zheng, Wu, Li *et al.* (2007 ▶); Xie *et al.* (2008 ▶); Chen & Li (2009 ▶). For comparative bond lengths, see: Allen *et al.* (1987 ▶). For hydrogen-bond motifs, see: Bernstein *et al.* (1995 ▶).
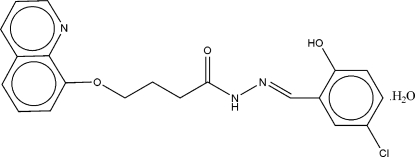


## Experimental

### 

#### Crystal data

C_20_H_18_ClN_3_O_3_·H_2_O
*M* *_r_* = 401.84
Monoclinic, 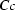

*a* = 11.167 (3) Å
*b* = 11.150 (3) Å
*c* = 30.909 (10) Å
β = 96.970 (12)°
*V* = 3820 (2) Å^3^
*Z* = 8
Mo *K*α radiation
μ = 0.23 mm^−1^
*T* = 295 K
0.32 × 0.15 × 0.10 mm

#### Data collection

Bruker SMART CCD area-detector diffractometer
Absorption correction: multi-scan (*SADABS*; Sheldrick, 1996 ▶) *T*_min_ = 0.929, *T*_max_ = 0.977
17043 measured reflections
5912 independent reflections
3796 reflections with *I* > 2σ(*I*)
*R*_int_ = 0.093

#### Refinement

*R*[*F* ^2^ > 2σ(*F* ^2^)] = 0.079
*wR*(*F* ^2^) = 0.198
*S* = 1.02
5912 reflections
508 parameters
2 restraints
H-atom parameters constrained
Δρ_max_ = 0.34 e Å^−3^
Δρ_min_ = −0.32 e Å^−3^
Absolute structure: Flack (1983), 2525 Friedel pairs
Flack parameter: 0.08 (13)

### 

Data collection: *SMART* (Bruker, 2007 ▶); cell refinement: *SAINT* (Bruker, 2007 ▶); data reduction: *SAINT*; program(s) used to solve structure: *SHELXS97* (Sheldrick, 2008 ▶); program(s) used to refine structure: *SHELXL97* (Sheldrick, 2008 ▶); molecular graphics: *SHELXTL* (Sheldrick, 2008 ▶); software used to prepare material for publication: *SHELXTL*.

## Supplementary Material

Crystal structure: contains datablocks global, I. DOI: 10.1107/S1600536809023733/pk2173sup1.cif

Structure factors: contains datablocks I. DOI: 10.1107/S1600536809023733/pk2173Isup2.hkl

Additional supplementary materials:  crystallographic information; 3D view; checkCIF report

## Figures and Tables

**Table 1:** Hydrogen-bond geometry (Å, °)

*D*—H⋯*A*	*D*—H	H⋯*A*	*D*⋯*A*	*D*—H⋯*A*
N2—H2⋯O7^i^	0.86	2.07	2.836 (9)	147
N5—H5⋯O8^ii^	0.86	2.05	2.820 (9)	149
O3—H3⋯N3	0.82	1.92	2.630 (8)	144
O6—H6⋯N6	0.82	1.91	2.633 (9)	147
O7—H29⋯N1	0.85	2.05	2.876 (8)	165
O7—H30⋯O5^iii^	0.85	2.06	2.839 (9)	153
O8—H31⋯N4	0.85	2.04	2.872 (9)	166
O8—H32⋯O2^iv^	0.85	1.99	2.845 (9)	180
C7—H7⋯O6^iii^	0.93	2.53	3.267 (10)	137
C27—H27⋯O3^v^	0.93	2.56	3.303 (10)	137
C19—Cl1⋯*Cg*5^vi^	1.74 (1)	3.63 (1)	4.127 (9)	94 (1)
C39—Cl2⋯*Cg*1^vii^	1.76 (1)	3.62 (1)	4.109 (9)	93 (1)

Symmetry codes: (i) 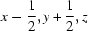; (ii) 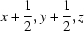; (iii) 
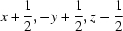
; (iv) 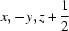; (v) 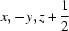; (vi) 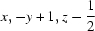; (vii) 
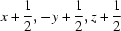
. *Cg1* and *Cg2* are the centroids of the N1/C8/C7/C6/C5/C9 and N4/C28/C27/C26/C25/C29 rings, respectively.

**Fig. 1.**
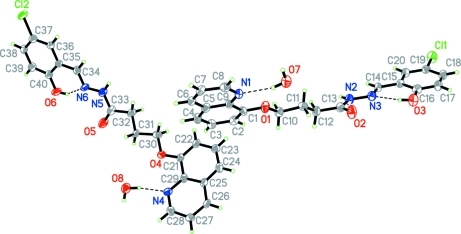
The molecular structure, with displacement ellipsoids at the 30% probability level.

**Fig. 2.**
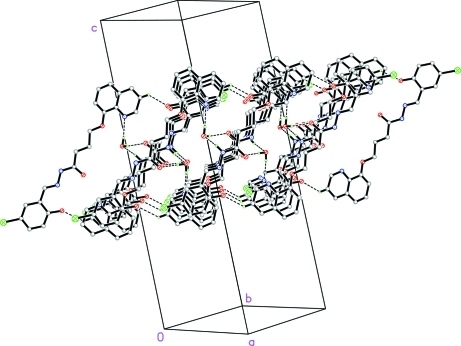
Part of the crystal structure showing hydrogen bonds as dashed lines. H atoms, except for those involved in hydrogen bonds, are not included.

## supplementary crystallographic information

### Comment

The rational construction of new matallosupramolecular architectures using logical combinations of rigid linear and angular components, has been the subject of much study during the last decade (Muraoka *et al.*,1998; Cai *et al.*, 2003; Pallavicini *et al.*, 2007). Most commonly, nitrogen heterocycles have been used to provide donors for coordination to metals within these assemblies, with pyridine rings being by far the most frequently used. More recently, flexible ligands have been employed to obtain access to topologies that are not available using more rigid ligands. Such flexibility can be introduced by means of combinations of methylene, ether, or thioether spacer groups between the donor sites, which permit the ligand to exist in various combinations as a result of rotations about single bonds. 8-Hydroxyquinoline and its derivatives are among the most extensively investigated ligands in this area (Xu *et al.*, 2002; Cai *et al.*, 2003; Chen *et al.*, 2005; Park *et al.*, 2006; Karmakar *et al.*, 2007; Zhang *et al.*, 2005; Wen *et al.*, 2005, Wei *et al.*, 2004; Zheng, Li *et al.*, 2008). In this contribution, we present the synthesis and crystal structure of a new ligand, which contains oxygen and nitrogen donors and flexible aliphatic spacers.

The bond lengths and angles are in good agreement with expected values (Allen *et al.*, 1987) and are comparable to those in the related compounds (Zheng, Wu, Lu *et al.*, 2006; Zheng, 2006; Zheng, Wu, Li *et al.*, 2007; Xie *et al.*, 2008; Chen & Li, 2009). X-ray crystallography reveals that the title compound was twinned by a 2-fold rotation about (100).  The crystals contain two crystallographically independent molecules with similar conformations, and two water molecules. The conformation along the C1—O1—C10—C11—C12—C13—N2—N3—C14—C15 and C21—O4—C30—C31—C32—C33—N5—N6—C34—C35 bond sequence are all *trans* (Fig.1). The C14—N3 and C34—N6 bond lengths of 1.290 (9) and 1.283 (9) Å respectively, indicate the presence of a typical C=N. The CN—N angle of 115.7 (6) and 116.2 (6)° are significantly smaller than the ideal value of 120° expected for *sp*^2^-hybridized N atoms and the dihedral angles between the benzene ring and quinoline ring system in the two molecules are 52.5 (7) and 53.9 (7)°. This is probably a consequence of repulsion between the nitrogen lone pairs and the adjacent N atom (Zheng, Qiu *et al.*, 2006). All torsion angles involving non-H atoms are close to 180°, which indicates that the molecules are essentially planar with the C=N bond adjacent to the benzene ring and quinoline group adopting a *trans* configuration with respect to its substitution. In the crystal packing, intramolecular O—H···N hydrogen bonds produce S(6) ring motifs (Bernstein *et al.*,1995) and there are also significant π-stacking interactions between the planar sections associated with the benzene ring and quinoline group. The organic molecules aggregate *via* intermolecular weak C—Cl···π and π–π interactions between the benzene ring and quinoline rings [centroid-centroid distances in the range of 3.696 (5)–3.892 (5) Å] and weak C—H···O contacts into an array of parallel sheets, and these layers are further linked by water molecules *via* N—H···O and O—H···O hydrogen bonds into a supramolecular two dimensional network (Fig. 2 and Table 1).

### Experimental

Reagents and solvents were of commercially available quality. The title complex was synthesized according to the method of Zheng, Li *et al.* 2008. 2-(quinolin-8-yloxy)butanehydrazide (0.01 mol), 5-chloro-2-hydroxybenzaldehyde (0.01 mol), ethanol (40 ml) and some drops of acetic acid were added to a 100 ml flask and refluxed for 6 h. After cooling to room temperature, the solid product was separated by filtration. Yellow single crystals suitable for X-ray diffraction were obtained by slow evaporation of a tetrahydrofuran solution over a period of 2 d.

### Refinement

All H atoms were placed in idealized positions (C—H = 0.93–0.97 Å, N—H = 0.86 Å, O—H = 0.82–0.85 Å) and refined as riding atoms with *U*_iso_(H) = 1.2*U*_eq_(C,*N*) and with *U*_iso_(H) = 1.5*U*_eq_(O).

### Crystal data

**Table:**

C_20_H_18_ClN_3_O_3_·H_2_O	*F*(000) = 1680
*M**_r_* = 401.84	*D*_x_ = 1.397 Mg m^−^^3^
Monoclinic, *C**c*	Mo *K*α radiation, λ = 0.71073 Å
Hall symbol: C -2yc	Cell parameters from 2234 reflections
*a* = 11.167 (3) Å	θ = 2.6–18.8°
*b* = 11.150 (3) Å	µ = 0.23 mm^−^^1^
*c* = 30.909 (10) Å	*T* = 295 K
β = 96.970 (12)°	Block, yellow
*V* = 3820 (2) Å^3^	0.32 × 0.15 × 0.10 mm
*Z* = 8	

### Data collection

**Table:**

Bruker SMART CCD area-detector diffractometer	5912 independent reflections
Radiation source: fine-focus sealed tube	3796 reflections with *I* > 2σ(*I*)
graphite	*R*_int_ = 0.093
φ and ω scans	θ_max_ = 25.0°, θ_min_ = 1.3°
Absorption correction: multi-scan (*SADABS*; Sheldrick, 1996)	*h* = −13→13
*T*_min_ = 0.929, *T*_max_ = 0.977	*k* = −12→13
17043 measured reflections	*l* = −32→36

### Refinement

**Table:**

Refinement on *F*^2^	Secondary atom site location: difference Fourier map
Least-squares matrix: full	Hydrogen site location: inferred from neighbouring sites
*R*[*F*^2^ > 2σ(*F*^2^)] = 0.079	H-atom parameters constrained
*wR*(*F*^2^) = 0.198	*w* = 1/[σ^2^(*F*_o_^2^) + (0.08*P*)^2^ + 4.5*P*] where *P* = (*F*_o_^2^ + 2*F*_c_^2^)/3
*S* = 1.02	(Δ/σ)_max_ < 0.001
5912 reflections	Δρ_max_ = 0.34 e Å^−^^3^
508 parameters	Δρ_min_ = −0.31 e Å^−^^3^
2 restraints	Absolute structure: Flack (1983), 2525 Friedel pairs
Primary atom site location: structure-invariant direct methods	Flack parameter: 0.08 (13)

### Special details

**Table:**

Geometry. All e.s.d.'s (except the e.s.d. in the dihedral angle between two l.s. planes) are estimated using the full covariance matrix. The cell e.s.d.'s are taken into account individually in the estimation of e.s.d.'s in distances, angles and torsion angles; correlations between e.s.d.'s in cell parameters are only used when they are defined by crystal symmetry. An approximate (isotropic) treatment of cell e.s.d.'s is used for estimating e.s.d.'s involving l.s. planes.
Refinement. Refinement of *F*^2^ against ALL reflections. The weighted *R*-factor *wR* and goodness of fit *S* are based on *F*^2^, conventional *R*-factors *R* are based on *F*, with *F* set to zero for negative *F*^2^. The threshold expression of *F*^2^ > σ(*F*^2^) is used only for calculating *R*-factors(gt) *etc*. and is not relevant to the choice of reflections for refinement. *R*-factors based on *F*^2^ are statistically about twice as large as those based on *F*, and *R*- factors based on ALL data will be even larger.

### Fractional atomic coordinates and isotropic or equivalent isotropic displacement parameters (Å^2^)

**Table:**

	*x*	*y*	*z*	*U*_iso_*/*U*_eq_	
Cl1	0.3021 (2)	0.92993 (19)	−0.10155 (8)	0.0771 (7)	
Cl2	1.56704 (19)	0.7328 (2)	0.70372 (8)	0.0731 (7)	
N1	1.1629 (5)	0.0908 (5)	0.18110 (18)	0.0446 (15)	
N2	0.5924 (6)	0.4429 (6)	0.0353 (2)	0.0564 (17)	
H2	0.5800	0.4886	0.0568	0.068*	
N3	0.5489 (5)	0.4771 (6)	−0.0078 (2)	0.0513 (16)	
N4	0.6334 (6)	−0.0301 (6)	0.4222 (2)	0.0503 (16)	
N5	1.0354 (6)	0.4892 (6)	0.5686 (2)	0.0535 (17)	
H5	1.0744	0.5082	0.5472	0.064*	
N6	1.0823 (5)	0.5171 (6)	0.6110 (2)	0.0520 (17)	
O1	0.9426 (4)	0.1745 (5)	0.16115 (17)	0.0575 (14)	
O2	0.6701 (6)	0.2718 (6)	0.0137 (2)	0.0782 (19)	
O3	0.4881 (6)	0.4409 (5)	−0.09162 (19)	0.0696 (17)	
H3	0.5252	0.4267	−0.0676	0.084*	
O4	0.7248 (5)	0.1826 (4)	0.44221 (16)	0.0518 (13)	
O5	0.8704 (6)	0.4003 (6)	0.59010 (19)	0.079 (2)	
O6	1.0749 (5)	0.5481 (6)	0.69511 (19)	0.0669 (16)	
H6	1.0486	0.5333	0.6697	0.100*	
O7	1.1208 (5)	0.1480 (5)	0.08981 (17)	0.0670 (16)	
H29	1.1252	0.1199	0.1155	0.100*	
H30	1.1950	0.1587	0.0878	0.100*	
O8	0.7190 (5)	−0.0214 (6)	0.51354 (18)	0.0719 (17)	
H31	0.6910	−0.0115	0.4869	0.108*	
H32	0.7045	−0.0963	0.5137	0.108*	
C1	0.9739 (7)	0.1482 (7)	0.2040 (2)	0.0491 (19)	
C2	0.9014 (7)	0.1634 (7)	0.2365 (3)	0.052 (2)	
H2A	0.8245	0.1956	0.2299	0.062*	
C3	0.9426 (7)	0.1306 (7)	0.2795 (3)	0.0494 (19)	
H3A	0.8926	0.1405	0.3012	0.059*	
C4	1.0553 (7)	0.0845 (7)	0.2896 (2)	0.054 (2)	
H4	1.0814	0.0616	0.3182	0.065*	
C5	1.1329 (6)	0.0711 (7)	0.2572 (2)	0.0494 (19)	
C6	1.2504 (7)	0.0246 (7)	0.2659 (3)	0.0471 (18)	
H6A	1.2801	0.0017	0.2941	0.057*	
C7	1.3212 (7)	0.0129 (7)	0.2331 (2)	0.050 (2)	
H7	1.3993	−0.0172	0.2384	0.060*	
C8	1.2709 (7)	0.0487 (7)	0.1908 (3)	0.053 (2)	
H8	1.3189	0.0413	0.1683	0.064*	
C9	1.0930 (6)	0.1029 (6)	0.2136 (2)	0.0402 (17)	
C10	0.8271 (7)	0.2281 (7)	0.1500 (2)	0.0493 (19)	
H10A	0.8222	0.3022	0.1661	0.059*	
H10B	0.7647	0.1744	0.1577	0.059*	
C11	0.8075 (7)	0.2541 (7)	0.1009 (2)	0.051 (2)	
H11A	0.8709	0.3061	0.0930	0.061*	
H11B	0.8095	0.1799	0.0847	0.061*	
C12	0.6870 (7)	0.3139 (8)	0.0903 (2)	0.058 (2)	
H12A	0.6257	0.2627	0.1003	0.070*	
H12B	0.6876	0.3885	0.1065	0.070*	
C13	0.6523 (7)	0.3405 (8)	0.0430 (3)	0.056 (2)	
C14	0.4998 (7)	0.5818 (7)	−0.0120 (2)	0.0495 (19)	
H14	0.4960	0.6297	0.0125	0.059*	
C15	0.4508 (6)	0.6247 (6)	−0.0543 (2)	0.0401 (16)	
C16	0.4452 (7)	0.5569 (7)	−0.0923 (3)	0.055 (2)	
C17	0.3957 (8)	0.6009 (7)	−0.1314 (3)	0.055 (2)	
H17	0.3913	0.5530	−0.1562	0.067*	
C18	0.3512 (7)	0.7182 (8)	−0.1345 (3)	0.057 (2)	
H18	0.3180	0.7498	−0.1611	0.068*	
C19	0.3579 (6)	0.7847 (7)	−0.0974 (3)	0.0482 (19)	
C20	0.4057 (7)	0.7447 (7)	−0.0576 (2)	0.0503 (19)	
H20	0.4091	0.7938	−0.0331	0.060*	
C21	0.6857 (6)	0.1661 (7)	0.3997 (2)	0.0416 (17)	
C22	0.6908 (7)	0.2486 (7)	0.3667 (2)	0.0519 (19)	
H22	0.7218	0.3249	0.3731	0.062*	
C23	0.6492 (7)	0.2178 (8)	0.3234 (3)	0.052 (2)	
H23	0.6553	0.2735	0.3014	0.063*	
C24	0.6010 (7)	0.1103 (8)	0.3132 (3)	0.056 (2)	
H24	0.5733	0.0922	0.2843	0.067*	
C25	0.5925 (7)	0.0266 (7)	0.3452 (2)	0.0475 (18)	
C26	0.5430 (7)	−0.0906 (7)	0.3365 (3)	0.0495 (19)	
H26	0.5114	−0.1116	0.3083	0.059*	
C27	0.5422 (8)	−0.1723 (7)	0.3701 (3)	0.055 (2)	
H27	0.5105	−0.2488	0.3647	0.066*	
C28	0.5896 (7)	−0.1386 (8)	0.4122 (3)	0.055 (2)	
H28	0.5906	−0.1951	0.4344	0.066*	
C29	0.6356 (6)	0.0495 (8)	0.3891 (2)	0.0471 (19)	
C30	0.7820 (7)	0.2949 (7)	0.4533 (3)	0.054 (2)	
H30A	0.7255	0.3599	0.4458	0.065*	
H30B	0.8501	0.3055	0.4370	0.065*	
C31	0.8249 (7)	0.2981 (7)	0.5017 (2)	0.0491 (19)	
H31A	0.7567	0.2889	0.5181	0.059*	
H31B	0.8805	0.2325	0.5093	0.059*	
C32	0.8867 (8)	0.4158 (7)	0.5129 (3)	0.058 (2)	
H32A	0.8319	0.4805	0.5031	0.070*	
H32B	0.9564	0.4222	0.4971	0.070*	
C33	0.9273 (7)	0.4315 (8)	0.5609 (3)	0.055 (2)	
C34	1.1874 (7)	0.5652 (7)	0.6160 (2)	0.051 (2)	
H34	1.2261	0.5797	0.5916	0.061*	
C35	1.2463 (6)	0.5970 (6)	0.6576 (2)	0.0432 (17)	
C36	1.1891 (7)	0.5900 (7)	0.6968 (2)	0.0480 (19)	
C37	1.2494 (7)	0.6258 (8)	0.7354 (3)	0.057 (2)	
H37	1.2118	0.6202	0.7606	0.068*	
C38	1.3654 (7)	0.6702 (7)	0.7381 (2)	0.0488 (19)	
H38	1.4055	0.6960	0.7646	0.059*	
C39	1.4200 (7)	0.6753 (7)	0.7010 (3)	0.054 (2)	
C40	1.3609 (7)	0.6448 (7)	0.6612 (3)	0.0502 (19)	
H40	1.3985	0.6563	0.6363	0.060*	

### Atomic displacement parameters (Å^2^)

**Table:**

	*U*^11^	*U*^22^	*U*^33^	*U*^12^	*U*^13^	*U*^23^
Cl1	0.0964 (18)	0.0496 (12)	0.0808 (16)	0.0246 (13)	−0.0072 (13)	0.0021 (11)
Cl2	0.0459 (11)	0.0978 (17)	0.0739 (14)	−0.0247 (12)	0.0002 (10)	−0.0134 (13)
N1	0.045 (4)	0.049 (4)	0.038 (4)	0.004 (3)	0.000 (3)	0.001 (3)
N2	0.055 (4)	0.057 (4)	0.055 (4)	0.015 (3)	−0.004 (3)	0.001 (3)
N3	0.042 (4)	0.054 (4)	0.055 (4)	0.010 (3)	−0.003 (3)	0.008 (3)
N4	0.050 (4)	0.043 (4)	0.056 (4)	−0.008 (3)	0.000 (3)	−0.004 (3)
N5	0.059 (4)	0.057 (4)	0.044 (4)	−0.010 (3)	0.002 (3)	−0.017 (3)
N6	0.041 (4)	0.057 (4)	0.055 (4)	−0.016 (3)	−0.004 (3)	−0.007 (3)
O1	0.040 (3)	0.083 (4)	0.049 (3)	0.017 (3)	0.000 (2)	0.002 (3)
O2	0.108 (5)	0.067 (4)	0.057 (4)	0.024 (4)	−0.003 (4)	−0.006 (3)
O3	0.096 (5)	0.045 (3)	0.064 (4)	0.025 (3)	−0.007 (3)	−0.009 (3)
O4	0.065 (3)	0.043 (3)	0.045 (3)	−0.016 (3)	0.001 (3)	−0.005 (2)
O5	0.064 (4)	0.126 (6)	0.048 (4)	−0.026 (4)	0.008 (3)	−0.018 (4)
O6	0.048 (3)	0.082 (4)	0.070 (4)	−0.026 (3)	0.009 (3)	−0.002 (4)
O7	0.066 (4)	0.078 (4)	0.056 (4)	0.006 (3)	0.003 (3)	−0.004 (3)
O8	0.067 (4)	0.100 (5)	0.049 (3)	−0.013 (3)	0.007 (3)	−0.001 (3)
C1	0.046 (5)	0.057 (5)	0.044 (5)	−0.001 (4)	0.000 (4)	0.015 (4)
C2	0.047 (4)	0.051 (5)	0.058 (5)	0.004 (4)	0.009 (4)	−0.010 (4)
C3	0.045 (5)	0.055 (5)	0.049 (5)	0.008 (4)	0.010 (4)	−0.001 (4)
C4	0.058 (5)	0.070 (5)	0.034 (4)	0.005 (4)	0.006 (4)	−0.002 (4)
C5	0.037 (4)	0.063 (5)	0.048 (5)	−0.001 (4)	0.003 (3)	−0.004 (4)
C6	0.043 (4)	0.049 (5)	0.048 (5)	−0.002 (4)	0.000 (4)	0.003 (4)
C7	0.043 (4)	0.056 (5)	0.049 (5)	0.000 (4)	−0.001 (4)	−0.013 (4)
C8	0.049 (5)	0.044 (4)	0.068 (6)	0.000 (4)	0.011 (4)	−0.002 (4)
C9	0.045 (4)	0.036 (4)	0.039 (4)	0.001 (3)	0.003 (3)	0.002 (3)
C10	0.043 (4)	0.046 (4)	0.056 (5)	0.014 (4)	−0.002 (4)	0.001 (4)
C11	0.050 (5)	0.039 (4)	0.063 (5)	0.006 (4)	0.010 (4)	0.007 (4)
C12	0.060 (5)	0.062 (5)	0.048 (5)	0.005 (4)	−0.010 (4)	0.004 (4)
C13	0.058 (5)	0.048 (5)	0.058 (6)	0.005 (4)	−0.007 (4)	0.007 (4)
C14	0.051 (4)	0.054 (5)	0.042 (4)	0.002 (4)	0.001 (3)	−0.003 (4)
C15	0.032 (4)	0.045 (4)	0.042 (4)	0.006 (3)	0.001 (3)	−0.004 (3)
C16	0.051 (5)	0.047 (5)	0.066 (6)	0.010 (4)	0.006 (4)	−0.003 (4)
C17	0.065 (5)	0.059 (5)	0.042 (5)	0.008 (4)	0.006 (4)	−0.012 (4)
C18	0.045 (5)	0.070 (6)	0.052 (5)	0.006 (4)	−0.008 (4)	0.006 (4)
C19	0.041 (4)	0.050 (5)	0.053 (5)	0.000 (3)	0.001 (4)	0.000 (4)
C20	0.053 (5)	0.048 (4)	0.050 (5)	0.006 (4)	0.004 (4)	−0.002 (4)
C21	0.038 (4)	0.053 (5)	0.033 (4)	−0.001 (3)	0.003 (3)	−0.005 (4)
C22	0.055 (5)	0.051 (5)	0.046 (5)	−0.003 (4)	−0.007 (4)	−0.008 (4)
C23	0.054 (5)	0.052 (5)	0.052 (5)	−0.005 (4)	0.009 (4)	−0.007 (4)
C24	0.048 (4)	0.078 (6)	0.043 (5)	0.006 (4)	0.002 (3)	0.007 (4)
C25	0.045 (4)	0.050 (5)	0.046 (5)	0.001 (4)	−0.005 (3)	−0.004 (4)
C26	0.050 (5)	0.053 (5)	0.042 (4)	−0.002 (4)	−0.010 (3)	−0.007 (4)
C27	0.071 (6)	0.035 (4)	0.058 (6)	−0.005 (4)	0.006 (4)	−0.012 (4)
C28	0.061 (5)	0.062 (5)	0.043 (5)	0.001 (4)	0.009 (4)	0.006 (4)
C29	0.035 (4)	0.073 (6)	0.033 (4)	0.004 (4)	0.005 (3)	0.005 (4)
C30	0.042 (4)	0.043 (5)	0.078 (6)	0.000 (3)	0.012 (4)	0.001 (4)
C31	0.047 (4)	0.054 (5)	0.043 (5)	−0.006 (4)	−0.009 (3)	−0.008 (4)
C32	0.059 (5)	0.058 (5)	0.055 (5)	−0.012 (4)	−0.002 (4)	−0.014 (4)
C33	0.046 (5)	0.068 (6)	0.050 (5)	0.005 (4)	0.005 (4)	−0.014 (4)
C34	0.049 (5)	0.062 (5)	0.042 (5)	0.000 (4)	0.004 (4)	0.001 (4)
C35	0.044 (4)	0.038 (4)	0.047 (4)	−0.003 (3)	0.002 (3)	−0.003 (3)
C36	0.050 (5)	0.044 (4)	0.049 (5)	0.004 (3)	0.004 (4)	0.000 (4)
C37	0.057 (5)	0.074 (6)	0.040 (5)	−0.004 (4)	0.010 (4)	−0.003 (4)
C38	0.042 (4)	0.063 (5)	0.041 (4)	0.003 (4)	0.004 (3)	−0.002 (4)
C39	0.049 (5)	0.062 (5)	0.050 (5)	−0.006 (4)	0.007 (4)	0.000 (4)
C40	0.050 (5)	0.055 (5)	0.045 (5)	−0.010 (4)	0.002 (4)	0.004 (4)

### Geometric parameters (Å, °)

**Table:**

Cl1—C19	1.735 (8)	C12—C13	1.495 (11)
Cl2—C39	1.756 (8)	C12—H12A	0.9700
N1—C8	1.295 (9)	C12—H12B	0.9700
N1—C9	1.352 (8)	C14—C15	1.439 (10)
N2—C13	1.329 (10)	C14—H14	0.9300
N2—N3	1.414 (9)	C15—C16	1.392 (10)
N2—H2	0.8600	C15—C20	1.428 (10)
N3—C14	1.290 (9)	C16—C17	1.358 (11)
N4—C28	1.328 (10)	C17—C18	1.398 (11)
N4—C29	1.357 (9)	C17—H17	0.9300
N5—C33	1.363 (10)	C18—C19	1.360 (11)
N5—N6	1.389 (8)	C18—H18	0.9300
N5—H5	0.8600	C19—C20	1.357 (10)
N6—C34	1.283 (9)	C20—H20	0.9300
O1—C1	1.359 (9)	C21—C22	1.380 (10)
O1—C10	1.426 (8)	C21—C29	1.438 (11)
O2—C13	1.221 (10)	C22—C23	1.403 (11)
O3—C16	1.378 (9)	C22—H22	0.9300
O3—H3	0.8200	C23—C24	1.336 (12)
O4—C21	1.346 (8)	C23—H23	0.9300
O4—C30	1.429 (9)	C24—C25	1.374 (11)
O5—C33	1.214 (9)	C24—H24	0.9300
O6—C36	1.353 (9)	C25—C29	1.405 (10)
O6—H6	0.8200	C25—C26	1.431 (11)
O7—H29	0.8498	C26—C27	1.383 (11)
O7—H30	0.8474	C26—H26	0.9300
O8—H31	0.8522	C27—C28	1.395 (11)
O8—H32	0.8505	C27—H27	0.9300
C1—C2	1.375 (10)	C28—H28	0.9300
C1—C9	1.420 (10)	C30—C31	1.514 (11)
C2—C3	1.401 (11)	C30—H30A	0.9700
C2—H2A	0.9300	C30—H30B	0.9700
C3—C4	1.361 (11)	C31—C32	1.504 (11)
C3—H3A	0.9300	C31—H31A	0.9700
C4—C5	1.410 (10)	C31—H31B	0.9700
C4—H4	0.9300	C32—C33	1.509 (11)
C5—C6	1.407 (10)	C32—H32A	0.9700
C5—C9	1.412 (9)	C32—H32B	0.9700
C6—C7	1.365 (10)	C34—C35	1.414 (10)
C6—H6A	0.9300	C34—H34	0.9300
C7—C8	1.418 (11)	C35—C40	1.377 (10)
C7—H7	0.9300	C35—C36	1.440 (10)
C8—H8	0.9300	C36—C37	1.359 (11)
C10—C11	1.534 (10)	C37—C38	1.380 (11)
C10—H10A	0.9700	C37—H37	0.9300
C10—H10B	0.9700	C38—C39	1.364 (11)
C11—C12	1.502 (10)	C38—H38	0.9300
C11—H11A	0.9700	C39—C40	1.367 (11)
C11—H11B	0.9700	C40—H40	0.9300
			
C8—N1—C9	118.1 (6)	C19—C18—H18	121.0
C13—N2—N3	120.5 (7)	C17—C18—H18	121.0
C13—N2—H2	119.7	C20—C19—C18	124.2 (7)
N3—N2—H2	119.7	C20—C19—Cl1	118.2 (6)
C14—N3—N2	115.7 (6)	C18—C19—Cl1	117.6 (6)
C28—N4—C29	117.4 (7)	C19—C20—C15	117.9 (7)
C33—N5—N6	119.6 (6)	C19—C20—H20	121.0
C33—N5—H5	120.2	C15—C20—H20	121.0
N6—N5—H5	120.2	O4—C21—C22	126.4 (7)
C34—N6—N5	116.2 (6)	O4—C21—C29	114.7 (6)
C1—O1—C10	116.4 (6)	C22—C21—C29	118.9 (7)
C16—O3—H3	109.5	C21—C22—C23	120.2 (7)
C21—O4—C30	115.9 (6)	C21—C22—H22	119.9
C36—O6—H6	109.5	C23—C22—H22	119.9
H29—O7—H30	100.0	C24—C23—C22	121.5 (8)
H31—O8—H32	95.0	C24—C23—H23	119.2
O1—C1—C2	125.5 (7)	C22—C23—H23	119.2
O1—C1—C9	113.9 (6)	C23—C24—C25	120.0 (8)
C2—C1—C9	120.6 (7)	C23—C24—H24	120.0
C1—C2—C3	120.4 (7)	C25—C24—H24	120.0
C1—C2—H2A	119.8	C24—C25—C29	121.8 (8)
C3—C2—H2A	119.8	C24—C25—C26	122.9 (7)
C4—C3—C2	120.4 (7)	C29—C25—C26	115.3 (7)
C4—C3—H3A	119.8	C27—C26—C25	119.8 (7)
C2—C3—H3A	119.8	C27—C26—H26	120.1
C3—C4—C5	120.5 (7)	C25—C26—H26	120.1
C3—C4—H4	119.8	C26—C27—C28	119.1 (7)
C5—C4—H4	119.8	C26—C27—H27	120.4
C6—C5—C4	122.9 (7)	C28—C27—H27	120.4
C6—C5—C9	117.0 (6)	N4—C28—C27	123.4 (7)
C4—C5—C9	120.1 (7)	N4—C28—H28	118.3
C7—C6—C5	120.3 (7)	C27—C28—H28	118.3
C7—C6—H6A	119.9	N4—C29—C25	124.9 (7)
C5—C6—H6A	119.9	N4—C29—C21	117.5 (6)
C6—C7—C8	117.3 (7)	C25—C29—C21	117.6 (7)
C6—C7—H7	121.4	O4—C30—C31	109.6 (6)
C8—C7—H7	121.4	O4—C30—H30A	109.7
N1—C8—C7	124.7 (7)	C31—C30—H30A	109.7
N1—C8—H8	117.7	O4—C30—H30B	109.7
C7—C8—H8	117.7	C31—C30—H30B	109.7
N1—C9—C5	122.7 (6)	H30A—C30—H30B	108.2
N1—C9—C1	119.3 (6)	C32—C31—C30	109.2 (6)
C5—C9—C1	118.0 (6)	C32—C31—H31A	109.8
O1—C10—C11	109.6 (6)	C30—C31—H31A	109.8
O1—C10—H10A	109.8	C32—C31—H31B	109.8
C11—C10—H10A	109.8	C30—C31—H31B	109.8
O1—C10—H10B	109.8	H31A—C31—H31B	108.3
C11—C10—H10B	109.8	C31—C32—C33	113.7 (7)
H10A—C10—H10B	108.2	C31—C32—H32A	108.8
C12—C11—C10	108.5 (6)	C33—C32—H32A	108.8
C12—C11—H11A	110.0	C31—C32—H32B	108.8
C10—C11—H11A	110.0	C33—C32—H32B	108.8
C12—C11—H11B	110.0	H32A—C32—H32B	107.7
C10—C11—H11B	110.0	O5—C33—N5	122.6 (7)
H11A—C11—H11B	108.4	O5—C33—C32	125.1 (7)
C13—C12—C11	114.9 (7)	N5—C33—C32	112.2 (7)
C13—C12—H12A	108.5	N6—C34—C35	122.2 (7)
C11—C12—H12A	108.5	N6—C34—H34	118.9
C13—C12—H12B	108.5	C35—C34—H34	118.9
C11—C12—H12B	108.5	C40—C35—C34	119.8 (7)
H12A—C12—H12B	107.5	C40—C35—C36	117.1 (7)
O2—C13—N2	122.0 (8)	C34—C35—C36	123.0 (7)
O2—C13—C12	123.7 (7)	O6—C36—C37	120.0 (7)
N2—C13—C12	114.2 (8)	O6—C36—C35	119.9 (7)
N3—C14—C15	120.2 (7)	C37—C36—C35	120.1 (7)
N3—C14—H14	119.9	C36—C37—C38	121.3 (7)
C15—C14—H14	119.9	C36—C37—H37	119.3
C16—C15—C20	118.1 (7)	C38—C37—H37	119.3
C16—C15—C14	124.1 (7)	C39—C38—C37	118.4 (8)
C20—C15—C14	117.8 (6)	C39—C38—H38	120.8
C17—C16—O3	117.2 (7)	C37—C38—H38	120.8
C17—C16—C15	121.7 (7)	C38—C39—C40	122.0 (7)
O3—C16—C15	121.1 (7)	C38—C39—Cl2	119.1 (6)
C16—C17—C18	120.1 (7)	C40—C39—Cl2	118.7 (6)
C16—C17—H17	119.9	C39—C40—C35	120.8 (7)
C18—C17—H17	119.9	C39—C40—H40	119.6
C19—C18—C17	118.0 (7)	C35—C40—H40	119.6
			
C13—N2—N3—C14	−175.2 (7)	C14—C15—C20—C19	−179.0 (7)
C33—N5—N6—C34	176.2 (7)	C30—O4—C21—C22	−3.2 (10)
C10—O1—C1—C2	3.4 (11)	C30—O4—C21—C29	176.2 (6)
C10—O1—C1—C9	−175.8 (6)	O4—C21—C22—C23	178.3 (7)
O1—C1—C2—C3	178.7 (7)	C29—C21—C22—C23	−1.0 (11)
C9—C1—C2—C3	−2.1 (11)	C21—C22—C23—C24	1.9 (12)
C1—C2—C3—C4	0.7 (12)	C22—C23—C24—C25	−0.7 (12)
C2—C3—C4—C5	1.2 (12)	C23—C24—C25—C29	−1.3 (12)
C3—C4—C5—C6	179.6 (7)	C23—C24—C25—C26	−179.4 (7)
C3—C4—C5—C9	−1.5 (12)	C24—C25—C26—C27	177.0 (8)
C4—C5—C6—C7	179.5 (7)	C29—C25—C26—C27	−1.2 (11)
C9—C5—C6—C7	0.7 (11)	C25—C26—C27—C28	0.2 (12)
C5—C6—C7—C8	−0.3 (11)	C29—N4—C28—C27	−2.6 (12)
C9—N1—C8—C7	0.6 (11)	C26—C27—C28—N4	1.7 (13)
C6—C7—C8—N1	−0.4 (12)	C28—N4—C29—C25	1.5 (11)
C8—N1—C9—C5	−0.3 (10)	C28—N4—C29—C21	−178.5 (7)
C8—N1—C9—C1	−179.6 (7)	C24—C25—C29—N4	−177.9 (7)
C6—C5—C9—N1	−0.4 (11)	C26—C25—C29—N4	0.3 (11)
C4—C5—C9—N1	−179.3 (7)	C24—C25—C29—C21	2.1 (11)
C6—C5—C9—C1	179.0 (7)	C26—C25—C29—C21	−179.7 (7)
C4—C5—C9—C1	0.1 (11)	O4—C21—C29—N4	−0.3 (9)
O1—C1—C9—N1	0.4 (10)	C22—C21—C29—N4	179.1 (6)
C2—C1—C9—N1	−178.9 (7)	O4—C21—C29—C25	179.7 (6)
O1—C1—C9—C5	−179.0 (6)	C22—C21—C29—C25	−0.9 (10)
C2—C1—C9—C5	1.7 (11)	C21—O4—C30—C31	−177.9 (6)
C1—O1—C10—C11	178.9 (6)	O4—C30—C31—C32	179.1 (6)
O1—C10—C11—C12	−178.3 (7)	C30—C31—C32—C33	176.8 (7)
C10—C11—C12—C13	−177.6 (7)	N6—N5—C33—O5	−1.8 (12)
N3—N2—C13—O2	−1.4 (12)	N6—N5—C33—C32	176.2 (7)
N3—N2—C13—C12	−177.1 (7)	C31—C32—C33—O5	−39.6 (12)
C11—C12—C13—O2	40.8 (12)	C31—C32—C33—N5	142.4 (7)
C11—C12—C13—N2	−143.5 (8)	N5—N6—C34—C35	−179.3 (7)
N2—N3—C14—C15	−179.0 (6)	N6—C34—C35—C40	177.8 (7)
N3—C14—C15—C16	4.3 (11)	N6—C34—C35—C36	−6.8 (12)
N3—C14—C15—C20	−175.3 (7)	C40—C35—C36—O6	177.6 (7)
C20—C15—C16—C17	−1.8 (11)	C34—C35—C36—O6	2.1 (11)
C14—C15—C16—C17	178.6 (8)	C40—C35—C36—C37	−2.2 (10)
C20—C15—C16—O3	179.4 (7)	C34—C35—C36—C37	−177.8 (7)
C14—C15—C16—O3	−0.3 (11)	O6—C36—C37—C38	−179.3 (7)
O3—C16—C17—C18	−179.5 (7)	C35—C36—C37—C38	0.6 (12)
C15—C16—C17—C18	1.6 (13)	C36—C37—C38—C39	−1.4 (12)
C16—C17—C18—C19	−0.9 (12)	C37—C38—C39—C40	4.0 (12)
C17—C18—C19—C20	0.5 (12)	C37—C38—C39—Cl2	179.4 (6)
C17—C18—C19—Cl1	−179.8 (6)	C38—C39—C40—C35	−5.9 (12)
C18—C19—C20—C15	−0.7 (12)	Cl2—C39—C40—C35	178.7 (6)
Cl1—C19—C20—C15	179.6 (5)	C34—C35—C40—C39	−179.5 (7)
C16—C15—C20—C19	1.3 (10)	C36—C35—C40—C39	4.8 (11)

### Hydrogen-bond geometry (Å, °)

**Table:**

*D*—H···*A*	*D*—H	H···*A*	*D*···*A*	*D*—H···*A*
N2—H2···O7^i^	0.86	2.07	2.836 (9)	147
N5—H5···O8^ii^	0.86	2.05	2.820 (9)	149
O3—H3···N3	0.82	1.92	2.630 (8)	144
O6—H6···N6	0.82	1.91	2.633 (9)	147
O7—H29···N1	0.85	2.05	2.876 (8)	165
O7—H30···O5^iii^	0.85	2.06	2.839 (9)	153
O8—H31···N4	0.85	2.04	2.872 (9)	166
O8—H32···O2^iv^	0.85	1.99	2.845 (9)	180
C7—H7···O6^iii^	0.93	2.53	3.267 (10)	137
C27—H27···O3^v^	0.93	2.56	3.303 (10)	137
C19—Cl1···Cg5^vi^	1.735 (8)	3.632 (4)	4.127 (9)	93.8 (3)
C39—Cl2···Cg1^vii^	1.756 (8)	3.616 (4)	4.109 (9)	93.3 (3)

Symmetry codes: (i) *x*−1/2, *y*+1/2, *z*; (ii) *x*+1/2, *y*+1/2, *z*; (iii) *x*+1/2, −*y*+1/2, *z*−1/2; (iv) *x*, −*y*, *z*+1/2; (v) *x*, −*y*, *z*+1/2; (vi) *x*, −*y*+1, *z*−1/2; (vii) *x*+1/2, −*y*+1/2, *z*+1/2.
